# Dual Roles of Autophagy in Radiation‐Induced Brain Injury: Mechanistic Insights and Therapeutic Implications

**DOI:** 10.1111/cns.70464

**Published:** 2025-06-09

**Authors:** Jiayu Tian, Yanna Mao, Dandan Liu, Tao Li, Lihuan Shi, Yafeng Wang, Changlian Zhu

**Affiliations:** ^1^ Henan Neurodevelopment Engineering Research Center for Children Children's Hospital Affiliated to Zhengzhou University, Henan Children's Hospital, Zhengzhou Children's Hospital Zhengzhou China; ^2^ Department of Hematology and Oncology Children's Hospital Affiliated to Zhengzhou University, Henan Children's Hospital, Zhengzhou Children's Hospital Zhengzhou China; ^3^ Department of Electrocardiogram Children's Hospital Affiliated to Zhengzhou University, Henan Children's Hospital, Zhengzhou Children's Hospital Zhengzhou China; ^4^ Henan Medical Key Laboratory of Pediatric Hematology Children's Hospital Affiliated to Zhengzhou University Zhengzhou China; ^5^ Henan Key Laboratory of Child Brain Injury and Henan Pediatric Clinical Research Center Institute of Neuroscience and Third Affiliated Hospital of Zhengzhou University Zhengzhou China; ^6^ Center for Brain Repair and Rehabilitation Institute of Neuroscience and Physiology, Sahlgrenska Academy, University of Gothenburg Göteborg Sweden

**Keywords:** autophagy, autophagy modulator, brain injury, neuroinflammation, radiation

## Abstract

**Background:**

Cranial radiotherapy, while essential for treating brain tumors, often leads to radiation‐induced brain injury, a debilitating condition marked by cognitive decline and neuronal damage. Autophagy, a key cellular process for recycling damaged organelles and proteins, has emerged as both a protective and detrimental player in radiation‐induced brain injury.

**Methods:**

This review systematically explores the dualistic role of autophagy in radiation‐induced brain injury, synthesizing insights on its interplay with apoptosis, ferroptosis, neuroinflammation, oxidative stress, the blood–brain barrier, mitophagy, endoplasmic reticulum stress, and mitochondrial biogenesis.

**Results:**

While autophagy supports neuronal resilience by mitigating oxidative and inflammatory stress, excessive or dysregulated autophagy can lead to autophagic cell death and exacerbate injury. Pharmacological modulators such as mTOR inhibitors, AMP‐activated protein kinase activators, demonstrate therapeutic potential in preclinical settings.

**Conclusion:**

By elucidating the mechanistic underpinnings of autophagy in radiation‐induced brain injury, this review underscores its dual roles and therapeutic relevance, offering a foundation for targeted interventions that optimize autophagic balance to protect brain function postradiotherapy.

## Graphical abstract

Under varying conditions, autophagy plays a dualistic role in radiation‐induced brain injury (RIBI), interacting intricately with apoptosis, ferroptosis, neuroinflammation, oxidative stress, blood–brain barrier (BBB) disruption, mitophagy, endoplasmic reticulum (ER) stress, and mitochondrial biogenesis. Modulation of autophagy has been shown to exert neuroprotective effects, highlighting its potential as a therapeutic target for the treatment of RIBI.

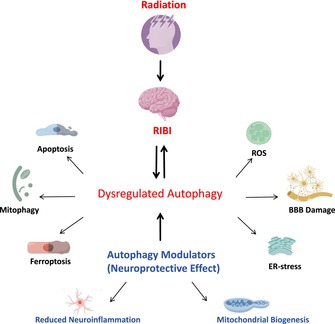

## 1 Introduction

Radiotherapy remains a cornerstone in the management of malignant brain tumors [1, 2], significantly improving patient survival through advancements such as stereotactic body radiation therapy (SBRT) and three‐dimensional conformal radiotherapy (3D‐CRT) [3]. However, radiation‐induced encephalopathy remains a frequent and debilitating adverse effect, manifesting in a substantial proportion of patients with clinical outcomes that include cognitive decline, cerebrovascular dysfunction, and a markedly reduced quality of life [4]. Despite its clinical significance, effective interventions to prevent or alleviate radiation‐induced brain injury are lacking, and the precise pathophysiological mechanisms underlying this condition remain incompletely understood.

Recent research has identified autophagy, a cellular process responsible for the degradation and recycling of damaged components, as a critical player in the body's response to stress and injury. Autophagy serves to maintain cellular homeostasis by removing damaged organelles and proteins, particularly under stress conditions such as radiation exposure [5]. Numerous recent studies indicate autophagy's involvement in regulating various mouse models of brain injury, suggesting that it may represent a potential therapeutic target for managing brain injury [6, 7, 8, 9]. Intriguingly, autophagy may function as a double‐edged sword: while it can protect neurons by alleviating cellular stress, excessive or dysregulated autophagy may exacerbate brain damage through autophagic cell death and inflammatory responses.

This review aims to explore the complex role of autophagy in radiation‐induced brain injury, focusing on its interactions with key cellular processes such as apoptosis, ferroptosis, neuroinflammation, reactive oxygen species (ROS) generation, and blood–brain barrier integrity. By examining these mechanisms, we seek to unravel the dual nature of autophagy and assess whether it can be harnessed as a therapeutic target. The modulation of autophagy through compounds such as mechanistic target of rapamycin (mTOR) inhibitors and AMP‐activated protein kinase (AMPK) activators offers promising potential for reducing radiation‐induced brain damage, but a deeper understanding of these pathways is crucial for developing safe and effective treatments.

By investigating the latest advances in autophagy research, this review aims to provide new insights into the prevention and treatment of radiation‐induced brain injury, ultimately offering hope for improving the prognosis and quality of life of patients undergoing cranial radiotherapy.

## 2 Regulation of Autophagy

Autophagy is a fundamental cellular process that promotes survival by removing damaged organelles and misfolded proteins, particularly under stress conditions such as nutrient deprivation, oxidative stress, and radiation exposure. Through this tightly regulated mechanism, cells maintain homeostasis and resilience [10, 11]. However, the regulation of autophagy is highly complex, involving numerous signaling pathways and molecular regulators that ensure a balance between protective and destructive outcomes [12]. In the setting of radiation‐induced brain injury, understanding the regulation of autophagy is essential, as its dysregulation may contribute to both neuronal survival and neurodegeneration. Ionizing radiation persistently activates the AMPK/mTOR signaling axis, which can promote excessive autophagy, resulting in nonselective degradation of mitochondria and cellular nutrients and ultimately exacerbating adenosine triphosphate (ATP) depletion and metabolic dysfunction [13]. Emerging evidence suggests that hyperactivated autophagy may trigger autophagic cell death via the BAX/BAK‐dependent mitochondrial apoptosis pathway. In irradiated brain tissue, the accumulation of autophagosomes has been observed alongside Caspase‐3 activation, indicating that these processes may act synergistically to aggravate neuronal loss [14].

The initiation and progression of autophagy are controlled by multiple proteins and signaling pathways, which coordinate the formation and function of autophagosomes—specialized vesicles that engulf and degrade cellular components. These processes involve a diverse range of proteins and kinases from the autophagy‐related protein family, which regulate the nucleation, elongation, and maturation of autophagosomes. These stages culminate in the fusion of autophagosomes with lysosomes, where the captured cellular components are degraded and recycled [15].

The autophagic regulatory network is primarily centered around the mammalian target of rapamycin complex 1 (mTORC1), a key suppressor of autophagy through kinase‐dependent signaling. Under homeostatic conditions, mTORC1 inhibits autophagy by phosphorylating UNC‐51‐like kinases 1 and 2 (ULK1/2), particularly at Ser757 on ULK1, thereby preventing initiation complex assembly [16, 17]. ULK1 serves as a critical autophagy initiator, and under cellular stressors such as ionizing radiation, the inhibition of mTORC1 relieves this suppression, allowing ULK1 to trigger autophagosome formation [18]. Moreover, AMPK, a sensor of cellular energy status, plays a key role in promoting autophagy by directly activating ULK1 and inhibiting mTORC1 [19].

Various pharmacological agents modulate autophagy by targeting mTOR and AMPK pathways. These modulators are of particular interest in the treatment of conditions involving cellular stress, such as radiation‐induced brain injury, where they can either promote or inhibit autophagy depending on the therapeutic goal. Drugs such as Metformin activate AMPK, which promotes autophagy [20]. AMPK acts as an energy sensor and, when activated, promotes autophagy by enhancing the energy‐conserving pathways within cells. AMPK activation has shown promise in reducing the damage caused by radiation by helping cells recover from oxidative stress and other forms of injury [21]. mTOR performs as an inhibitor of autophagy, and its suppression can stimulate autophagy initiation [22]. Furthermore, mTOR inhibitors including Rapamycin and Everolimus also activate the autophagy pathways [23]. Compounds such as MRT68921 target ULK1 directly, further promoting autophagy. By facilitating the initiation of autophagy, these agents help cells clear damaged organelles and maintain homeostasis.

Autophagy involves complex processes regulated by various proteins and pathways. Different forms of autophagy activate specific regulators, leading to distinct effects. For example, transcription factor EB (TFEB) activators like Trehalose stimulate autophagy [24, 25], while Torin1 and Torin2 inhibit mTOR and induce autophagy [26]. Lithium activates autophagy by increasing beclin‐1 expression [27], and HDACs like trichostatin A (TSA) and party suberoylanilide hydroxamic acid (SAHA) promote autophagy [28]. In contrast, Chloroquine (CQ) derivatives are effective autophagy inhibitors [29, 30], as is Wortmannin, which blocks PI3K and the Beclin‐1/PI3K complex [31]. Protein kinase B (PKB, also known as AKT) activation can reduce autophagy by counteracting mTOR inhibition [32, 33]. These agents offer potential for exploring autophagy mechanisms and therapeutic applications [34].

## 3 Autophagy and Radiation‐Induced Brain Injury

Radiation therapy is a common approach for treating primary and metastatic brain tumors, but it often leads to damage in brain tissue alongside the elimination of cancer cells [35]. Radiation‐induced brain injury is a frequent consequence, causing cognitive impairment in a majority of patients and significantly impacting the prognosis and quality of life of individuals with brain tumors [36]. Previous clinical trials and animal studies have demonstrated that hippocampal neurogenesis, neurovascular injury, and neuroinflammation resulting from radiation vary depending on dosage and age, as different components of brain tissue and the vascular system exhibit varying levels of radiosensitivity [37]. These findings underscore the importance of carefully designing and implementing radiation treatment plans to minimize such risks.

Radiation‐induced brain injury can be categorized based on the timing of clinical symptoms as acute, early delayed, and late delayed stages [38]. Acute injury manifests within hours or days of exposure, characterized by cerebral edema and microvascular changes such as blood–brain barrier disruption, vascular hyalinization, endothelial cell senescence, and fibrinoid necrosis [39]. Early delayed brain injury typically occurs weeks to months after irradiation and is associated with transient demyelination and impaired neural network [40, 41]. Late delayed brain injury usually occurs 6 months up to several years postexposure, involves significant vascular changes and glial cell damage, leading to substantial lesions in white and gray matter, cerebral perfusion deficits, and various levels of cognitive impairment that considerably impact patients' quality of life [41].

Autophagy is recognized as a significant cellular protective mechanism against radiation‐induced brain injury [42]. Radiation can damage DNA and cell membranes in brain tissue, triggering cellular signaling pathways that initiate autophagy [4]. This process involves the encapsulation of damaged organelles or proteins in autophagosomes, which are subsequently degraded and recycled after fusing with lysosomes. By doing so, autophagy helps restore normal cellular functions and maintain homeostasis [43]. Furthermore, autophagy counteracts inflammation by eliminating dysfunctional cells and abnormal proteins, thereby reducing the inflammatory cascade triggered by radiation exposure [44]. Moreover, it modulates immune cell activity, further inhibiting the progression of inflammatory responses [45].

However, autophagy can also have detrimental effects on radiation‐induced brain injury. Excessive autophagy may result in autophagic cell death, exacerbating brain damage [46]. Over‐activated autophagy could potentially impair synaptic plasticity by facilitating the degradation of synaptic vesicles [46, 47]. It may also influence the release of inflammatory cytokines and chemokines by modulating inflammasomes, which can cause further inflammation and tissue damage under certain conditions [48]. Radiation‐induced disruptions in energy metabolism may deplete intracellular nutrients, reducing cellular resilience and the capacity for recovery [49, 50].

Thus, the relationship between autophagy and radiation‐induced brain injury is complex and multifaceted. A thorough understanding of factors such as cell type, injury severity, autophagy status, and regulatory pathways is essential to explore autophagy's role in radiation‐induced brain injury. This knowledge will provide a theoretical foundation for preventing complications from radiotherapy and guide the development of new therapeutic strategies.

## 4 Mechanisms of Autophagy in Radiation‐Induced Brain Injury

### 4.1 Autophagy and Apoptosis

The interplay between autophagy and apoptosis in radiation‐induced brain injury is complex due to the cross‐regulation between these two cellular pathways of cell death. These pathways often interact with one another [51]. Some signaling pathways can regulate both autophagy and apoptosis, determining the fate of the cell [52]. For example, the iNOS/NF‐kB/NF‐IL6 and p53/Bax pathways are known to influence whether cells enter autophagy or apoptosis [36]. Another study demonstrated that Atg7‐mediated autophagy exerts a protective effect on neural stem cells by reducing radiation‐induced apoptosis [53]. The balance between these processes can be viewed as a critical determinant of cell survival or death [54].

Radiation‐induced brain injury triggers intracellular stress, leading to DNA damage and oxidative stress. When the stress is severe or prolonged, cells may undergo apoptosis. Under less extreme conditions, autophagy can play a protective role by removing damaged molecules and helping neuronal cells maintain stability [53, 55]. However, excessive autophagy can exacerbate apoptosis, particularly through the enhancement of neuroinflammation following irradiation [56, 57].

Autophagy plays a dual role in apoptosis regulation. For instance, Kaempferol has been shown to mediate the AMPK signaling pathway, promoting autophagy whilst inhibiting apoptosis, resulting in a protective effect against brain injury [13, 58]. The balance between autophagy and apoptosis is influenced by several factors, including cell type, the severity of injury, and the cell environment [59]. In conclusion, autophagy and apoptosis are key components of the cellular response to radiation‐induced brain injury, with their balance ultimately determining whether cells survive or perish.

### 4.2 Autophagy and Ferroptosis

In recent years, there has been extensive research on ferroptosis, a novel form of cellular death linked to intracellular iron disruption [60, 61]. Studies have demonstrated a correlation between autophagy and ferroptosis in the cellular response to radiation‐induced brain damage [62]. Ferroptosis may trigger autophagy due to excessive iron inducing oxidative stress and resulting in cellular damage [63]. When an excess of iron is present in a cell and ferroptosis is initiated, the autophagy pathway is activated by the cell to remove damaged components and organelles [64]. This autophagy pathway can mitigate the damage of ferroptosis to cells. Additionally, autophagy plays a key role in regulating iron metabolism and maintaining cellular homeostasis by eliminating abnormal iron metabolites. By doing so, autophagy can prevent ferroptosis [65], a process closely linked to oxidative stress induced by excess iron that stimulates the production of free radicals. Autophagy also helps to counteract oxidative stress by removing oxidation‐damaged molecules, thereby reducing cellular damage and promoting cell survival [66].

Ferroptosis is indeed an autophagy‐dependent form of cell death [67]. For example, knockout of BECN1 (beclin1) impairs erastin‐triggered ferroptosis by inhibiting autophagy [65]. Stimulator of stimulatory responses to cGAMP interactor 1 (STING1) also plays an important role in mediating the activation of autophagy [68]. The anti‐HIV drug zalcitabine induces mitochondrial DNA stress in pancreatic cancer cells, leading to activation of STING1‐mediated autophagy and autophagy‐dependent ferroptosis (ADF) [69]. We may be able to exploit these pathways as insights into the mechanisms underlying autophagy and ferroptosis induced by radiation.

### 4.3 Autophagy and Neuroinflammation

Neuroinflammation is an inflammatory response resulting from immune cell activation and cytokine release, which may occur in cases of radiation‐induced brain injury [70]. Autophagy could play a significant role in the onset and progression of neuroinflammation following radiation exposure. Conversely, neuroinflammation may influence the regulation of autophagy through mechanisms involving ROS, inflammasome, or ER stress‐related mitophagy [71, 72]. Autophagy helps to minimize cellular stress by removing damaged components and abnormal proteins, which in turn reduces the release of inflammatory cytokines and chemokines, ultimately inhibiting the onset of inflammation caused by radiation‐induced brain damage [48]. Radiation‐induced neuronal damage triggers the release of high‐mobility group box 1 (HMGB1) from neurons, which in turn activates microglia via TLR2/TLR4/RAGE receptors, driving neuroinflammation. HMGB1 nuclear translocation in neurons amplifies the production of pro‐inflammatory cytokines such as TNF‐α, IL‐6, further exacerbating neuronal apoptosis [73]. In addition, autophagy impacts not only the regulation of innate immune cell differentiation, degranulation, phagocytosis, and extracellular trap formation, but also influences phagocytosis, antigen presentation, cytokine production, and control of inflammasome activation [74, 75]. In radiation‐induced brain injury, autophagy affects the development of neuroinflammation by modulating the activity of immune cells [76].

### 4.4 Autophagy and ROS

ROS are highly reactive molecules generated by the partial reduction of oxygen molecules in cells. These include superoxide anions, hydrogen peroxide, and hydroxyl radicals. In the case of radiological brain injury, ROS generation leads to oxidative stress in neuronal cells, while autophagy plays a role in regulating ROS levels and mitigating cell damage [77, 78]. High levels of radiation exposure can cause an increase in intracellular ROS, triggering cellular stress responses. Autophagy works to mitigate ROS‐induced damage by removing damaged cellular components, proteins, and organelles, thus restoring a healthy oxidative cell balance within the cell [79]. This process supports cell survival following radiation‐induced brain damage. ROS can influence autophagy by regulating various signaling pathways, which help determine whether cells activate the autophagy pathway in response to oxidative stress. Inhibition of autophagy has been shown to suppress both radiation resistance and the associated activation of the Nrf2/HO‐1 signaling pathway. Furthermore, treatment with a ROS inhibitor blocked autophagy, downregulated Nrf2 and HO‐1 expression, and reduced radiation‐induced resistance [80]. However, excessive ROS levels may impair the autophagy process, leading to cellular dysfunction [81, 82].

### 4.5 Autophagy and Blood–Brain Barrier (BBB)

The BBB is a biological barrier consisting of cerebrovascular endothelial cells and tight junction proteins, which serve to limit the passage of compounds from the bloodstream into cerebral tissue [83]. Autophagy plays a crucial role in maintaining cellular homeostasis and responding to cellular stress, including in the cells of the BBB. Autophagy in BBB cells aids in the removal of damaged cellular components and proteins, such as abnormally aggregated claudin 5 in the cytosol and reduced expression of zonula occludens‐1, thus preserving cellular function and integrity [84]. Inflammatory responses induced by radiation‐induced brain injury can impair the integrity of the BBB by amplifying microvascular damage and upregulating the expression of leukocyte adhesion molecules, which result in increased permeability to harmful substances from the blood into the brain tissue [85]. In such cases, BBB cells can activate autophagy pathways to eliminate impaired cellular components and help sustain BBB function. Moreover, autophagy may provide a protective effect by clearing ROS and reducing BBB damage and inflammatory responses [86]. A study showed that radiotherapy inhibits autophagy while promoting oversecretion of vascular endothelial growth factor (VEGF) oversecretion in astrocytes by upregulating the phosphoinositide 3‐kinase (PI3K)‐AKT signaling pathway. Blocking the PI3K pathway has been shown to reduce VEGF oversecretion and mitigate BBB disruption, thereby alleviating BBB damage in patients receiving radiotherapy [87]. In addition, bone marrow mesenchymal stem cells, in combination with mannitol, exerted neuroprotective effects against radiation‐induced brain damage by regulating autophagy through the PI3K/AKT/mTOR signaling axis [88].

### 4.6 Mitophagy

Mitophagy is a distinct form of cellular autophagy that focuses on eliminating impaired or senescent mitochondria to preserve cellular energy metabolism and function [89]. Mitophagy may play significant roles in controlling cellular survival, damage, and repair as well as addressing oxidative stress during radiation‐induced brain injury [90]. Radiation induces oxidative stress, and mitochondria can contribute to the generation of oxidative stress [91]. Following radiation damage, mitophagy assists cells in adjusting to changes in energy metabolism by removing damaged mitochondria and reducing oxidative stress production. This ensures the maintenance of cellular oxidative homeostasis [20, 92]. In an investigation of radiation‐induced brain injury using an animal model, Atg7‐mediated mitochondrial autophagy inhibition was found to modulate the level of mitochondrial fission and fusion in neuronal cells. Such inhibition provided additional energy to myelination and reduced postirradiation cerebral white matter injury [93]. Urolithin A has been shown to enhance the expression of tight junction proteins in cultured endothelial cells through Pink1‐Parkin‐mediated mitophagy in irradiated astrocytes [94]. Therefore, the regulation of mitophagy helps maintain cellular homeostasis at a microscopic level and plays a crucial role in energy homeostasis in response to postirradiation damage [95].

### 4.7 Autophagy and Endoplasmic Reticulum (ER) Stress

Radiation‐induced ER stress can significantly impact mitochondrial metabolism [96]. The ER and mitochondria maintain functional coupling through mitochondria‐associated membranes (MAMs), which are essential for calcium homeostasis, lipid metabolism, and apoptotic signaling [97]. Radiation activates the unfolded protein response (UPR), leading to the stimulation of key ER stress sensors—including inositol‐requiring enzyme 1α (IRE1α), protein kinase RNA‐like ER kinase (PERK), and activating transcription factor 6 (ATF6)—which promote the abnormal release of calcium ions (Ca^2+^) from the ER into mitochondria [98]. This mitochondrial Ca^2+^ overload can cause a reduction in mitochondrial membrane potential (MMP), a surge in ROS, and impaired ATP production, thereby intensifying neuronal energy failure [99].

Moreover, ER‐stress inhibits protein synthesis through the PERK/eukaryotic translation initiation factor 2α (eIF2α) pathway and induces the proapoptotic transcription factor C/EBP homologous protein (CHOP) [100], ultimately triggering mitochondrial apoptosis through Bax/Bak‐mediated permeabilization of the mitochondrial membrane [101, 102]. ER stress also exacerbates mitochondrial DNA damage through activation of the ROS–NOD‐like receptor protein 3 (NLRP3) inflammasome axis, forming a vicious cycle that accelerates neuronal necrosis and glial activation [99, 103]. Collectively, these mechanisms highlight the pivotal role of ER stress in mediating oxidative damage and apoptotic signaling following radiation exposure.

### 4.8 Autophagy and Mitochondrial Biogenesis

Dysregulated mitochondrial biogenesis represents a critical contributor to impaired neuronal energy metabolism and secondary damage after radiation exposure. Radiation can directly damage mitochondrial DNA (mtDNA) or indirectly disrupt the biosynthetic regulatory network, thereby hindering mitochondrial repair and regeneration and exacerbating brain tissue pathology [104, 105]. Mitochondrial biogenesis is chiefly governed by the PGC‐1α/NRF‐1/TFAM signaling axis, involving the peroxisome proliferator‐activated receptor gamma coactivator‐1 alpha (PGC‐1α), nuclear respiratory factor 1 (NRF‐1), and mitochondrial transcription factor A (TFAM) [106, 107].

Radiation has been shown to significantly suppress PGC‐1α expression in neurons, leading to reduced transcriptional activity of downstream targets such as mitochondrial DNA polymerase gamma and TFAM, ultimately decreasing mtDNA copy number and mitochondrial protein synthesis [108]. This impaired biogenesis reduces ATP production and forces neurons to rely on glycolysis, resulting in lactate accumulation and intracellular acidosis—clinical features often observed as elevated lactate peaks in magnetic resonance spectroscopy (MRS) imaging of radiation‐injured brains [109, 110]. Additionally, insufficient mtDNA exacerbates dysfunction in electron transport chain complexes (e.g., complex IV), promoting electron leakage and ROS bursts that damage both mitochondrial and nuclear DNA, perpetuating a cycle of impaired biogenesis and oxidative stress [111]. Notably, preclinical studies indicate that pharmacological activation of PGC‐1α (e.g., with resveratrol) can enhance mtDNA replication and improve cognitive outcomes [112].

**FIGURE 1:**
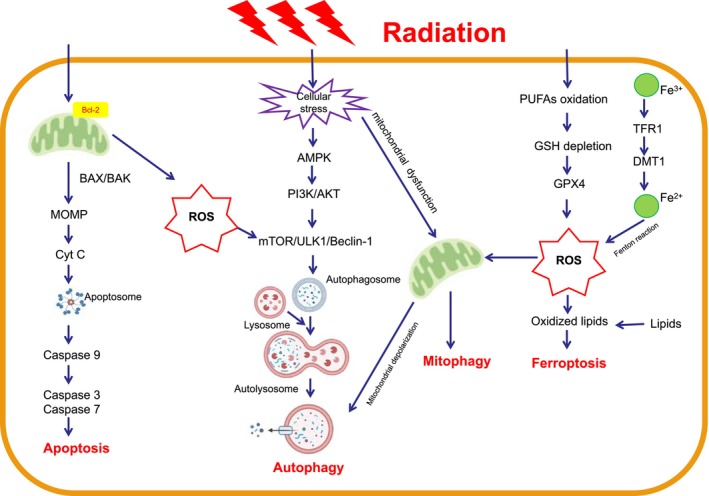
Illustrates various forms of neuronal cell death following exposure to radiation. After radiation exposure, both proapoptotic and antiapoptotic members of the Bcl‐2 family are activated. Of particular importance are Bax and Bak, which create pores in the mitochondrial membrane, leading to persistent and widespread mitochondrial outer membrane permeabilization (MOMP). This results in the release of mitochondrial proapoptotic factors, such as cytochrome C. Released cytochrome C combines with the caspase‐9 precursor to form an “apoptosome,” which then activates caspase‐9, ‐3, and ‐7, ultimately initiating the final steps of apoptosis. The classical autophagy pathway is primarily initiated through the interplay between the mTORC1 and ULK1 complexes. Upon radiation detection, AMPK inhibits the activity of the mTORC1 complex, relieving the suppression of autophagy. Subsequently, ULK1 is dephosphorylated and activated, promoting the formation of the ULK1 complex and contributing to the occurrence of autophagosomes. After autophagosomes encapsulate abnormal biological macromolecules or other cellular components, they fuse with lysosomes, leading to the degradation or recycling of the enclosed products. Ferroptosis, a form of cell death induced by iron toxicity, results from the accumulation of lipid peroxides, a process dependent on the generation of reactive oxygen species and the presence of iron following radiation exposure. The induction of ferroptosis after radiation exposure is accomplished through various pathways that affect the activity of GPXs, including GPX4, as well as the internal iron source from transferrin. AMPK, AMP‐Activated Protein Kinase; Cyt C, Cytochrome C; GPX4, Glutathione Peroxidase 4; MOMP, Mitochondrial Outer Membrane Permeabilization; mTORC1, Mammalian Target of Rapamycin Complex 1; ULK1, Unc‐51‐like Autophagy Activating Kinase 1.

Above all, the molecular mechanisms underlying autophagy and radiation‐induced brain injury are intricate, and their interrelationships remain complex (Figure 1). Conducting extensive research into the connection between these mechanisms and autophagy is crucial for comprehending the pathways involved in radiation‐induced brain injury and developing novel therapeutic strategies for its prevention.

## 5 Autophagy Modulator in the Treatment of Radiation‐Induced Brain Injury

**FIGURE 2:**
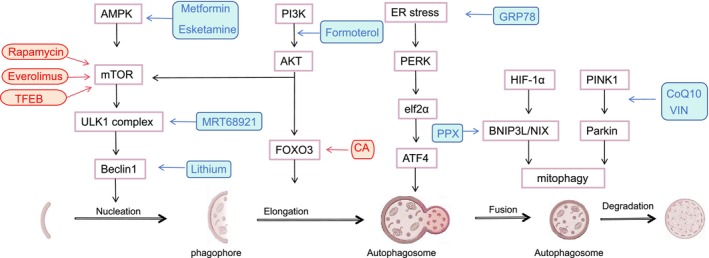
The signaling pathways and targets of autophagy regulators involved in brain injury. The inhibition of this pathway and its associated target is represented by a red arrow, whereas a blue arrow denotes activation. AMPK inhibits mTOR and subsequently triggers the downstream ULK1 complex, which in turn activates Beclin 1, thereby promoting the initiation of autophagy. The mTOR inhibitors rapamycin, everolimus, and TFEB have been identified. It has been demonstrated that esketamine and metformin activate AMPK, which in turn promotes autophagy. It has been proven that compounds such as MRT68921 target ULK1 directly, thereby further promoting autophagy. It has been shown that lithium activates autophagy by increasing the expression of Beclin‐1. Formoterol has been demonstrated to promote autophagy by acting on the PI3K/AKT/FOXO3 pathway, whereas CA has been shown to exert inhibitory effects on autophagy through this pathway. The ER chaperone GRP78 has been identified as a potential critical factor in the activation of autophagy. In the context of mitophagy, PPX, CoQ10, and VIN exert their effects by acting on the BNIP3L and PINK1/Parkin pathways, respectively, thereby activating autophagy. AKT, A serine/threonine kinase; AMPK, Adenosine 5′‐monophosphate‐activated protein kinase; ATF4, Activating transcription factor 4; BNIP3L, BCL2/adenovirus E1B 19 kDa interacting protein 3‐like; CA, Calysoin; CoQ10, Coenzyme Q10; eIF2α, Eukaryotic initiation factor‐2α; ER stress, Endoplasmic reticulum stress; FOXO3, Forkhead box O3; GRP78, Glucose‐regulated protein 78; HIF‐1α, Hypoxia‐inducible factor 1; mTOR, Mammalian target of rapamycin; Parkin, Parkin RBR E3 ubiquitin‐protein ligase; PERK, Protein kinase RNA (PKR)‐like ER kinase; PI3K, Phosphoinositide‐3‐kinase; PINK1, PTEN‐induced putative kinase 1; PPX, Pramipexole; ULK1, Unc‐51‐like kinase; VIN, Vinpocetine.

Significant progress has been made in the development and application of autophagy modulators [72]. A variety of drugs and compounds have been identified that can modulate different aspects of the autophagy pathway. Some of the autophagy regulators explored include mTOR inhibitors, AMPK activators, and TFEB activators [113, 114]. Figure 2 illustrates the signaling pathways and targets of autophagy regulators involved in brain injury. Furthermore, autophagy modulators offer the potential for greater selectivity and safety, providing more precise intervention in the autophagy pathway than conventional therapies, while minimizing adverse effects on healthy cells.

Autophagy regulators may influence the progression of radiation‐induced brain injury in several ways. Modulating autophagy at the cellular level can interrupt the development of radiation brain injury, offering a promising therapeutic approach. Previous research has shown that specific autophagy inhibitors can significantly mitigate radiation‐induced cerebral capillary damage, thereby prolonging the survival of zebrafish larvae [115]. This finding suggests that endothelial autophagy could be a potential target for preventing radiation‐induced brain toxicity.

Several preclinical and clinical trials have investigated the use of autophagy modulators in neurological injury‐related conditions (Table 1) and in radiation‐induced brain injury (Table 2) [23, 116, 117, 118, 119, 120, 121, 122, 123, 124, 125, 126, 127, 128, 129, 130, 131, 132], with particular focus on improving cognitive function, memory, and behavioral outcomes [133, 134]. However, the use of autophagy modulators specifically for radiation‐induced brain injury remains largely unexplored. It is important to recognize that the regulation of autophagy is complex, and responses may vary depending on cell type and disease context. As such, caution is needed when considering autophagy‐based therapeutic strategies, with attention to cell‐type specificity. Moreover, the use of autophagy modulators for treating radiation‐induced brain injury is still in the research phase, and further clinical studies are required to establish their efficacy and safety.

**TABLE 1:** The Clinical applications and mechanisms of autophagy regulators in brain injuries.

Modulators	Mechanisms	Clinical applications	References
Spermidine	Dissolution of amyloid‐beta plaques improve cognitive decline to trigger the process of autophagy	Alzheimer's disease	[116, 117]
PINK1‐mediated phosphorylation of the Parkin ubiquitin‐like domain	Primes mitochondrial translocation of Parkin and regulates mitophagy	Parkinson's disease	[118]
Chloroquine (CLQ)	p53‐dependent apoptotic activation and the inhibition of autophagic protein degradation	Radiation induced brain injury	[119]
Long‐term lithium treatment	Up‐regulation of neurotrophic response and autophagy, and downregulation of apoptosis, oxidative stress, and inflammation.	Alzheimer's disease and amyotrophic lateral sclerosis	[120]
High‐Dose Valproic acid	Improve Fatty acid and adenosine triphosphate (ATP) biosynthesis	Traumatic brain injury	[121]
Chloroquine	Inhibition of autophagy caused by radiotherapy and TMZ can ameliorate apoptosis in GBM cells	Radiation induced brain injury	[122]
Sirolimus (rapamycin)	mTOR inhibitor, promotes α‐synuclein autophagy	Multiple system atrophy, neurodegenerative disease	[123]
Felodipine	Upregulate autophagy which has been shown to be critical for the degradation of diverse intracytoplasmic aggregate‐prone proteins	Huntington's disease	[124]
Hypoxia‐preconditioned olfactory mucosa‐Mesenchymal stem cells	Improving microglia immune regulation and autophagy homeostasis in the SN, mediated by TGF‐β1 via activation of the ALK/PI3K/Akt signaling pathway in microglia	Parkinson's disease	[125]

**TABLE 2:** Autophagy modulator in the treatment of radiation induced brain injury.

Modulators	Mechanisms	References
Chloroquine (CLQ)	p53‐dependent apoptotic activation and the inhibition of autophagic protein degradation	[119]
Chloroquine	Inhibition of autophagy caused by radiotherapy and TMZ can ameliorate apoptosis in GBM cells	[122]
Melatonin	Increasing the release of neurotransmitters, antioxidants, anti‐inflammatory factors and reducing pro‐inflammatory cytokines and apoptosis in the brain of irradiated rats.	[126]
IR‐780 (A near‐infrared dye)	Improve cognitive dysfunction, reduce neuroinflammation, restore the expression of tight junction proteins, and reduce the levels of cellular reactive oxygen species and apoptosis.	[127]
Se@SiO2	Se@SiO2 NPs regulate the nuclear factor kappa B (NF‐κB) and mitogen‐activated protein kinase (MAPK) signaling pathways, thereby reduce the expression of inflammatory factors.	[128]
iPSC‐derived MSC (iMSC)	Suppress IR‐induced NFκB activation, TNF‐α release, and ROS production in THP1 cells. Increase expression of prosurvival factors, the PI3K/AKT/mTOR modulator PRAS40 and β‐catenin.	[129]
miR‐711	Attenuate degradation of Akt and Ang‐1 mRNAs and reduce intrinsic apoptosis, Inhibition of miR‐711 rescued Rad50 and Rad54l2 expression, enhancing DNA repair and reducing p53‐dependent apoptotic pathways.	[130]
Ginkgolide B (GB)	Reduce MST1 and thus apoptosis. Knockdown of the Netrin‐1 receptor DCC abolishes GB and mitigates the protective effects of X‐ray‐induced ROS production and apoptosis.	[131]
Long noncoding RNAs (lncRNAs)	Modulate the activation of NF‐κB/MAPK signaling and subsequent inflammatory cytokine secretion	[23]
Sildenafil (SD) and simvastatin (SV)	The coadministration of SV and SD offers a neuroprotective effect against irradiation‐induced brain injury due to its NO donor/BH4 regulatory activities, anti‐inflammatory and antioxidant properties that modulate IDO/KYN pathway.	[132]

## 6 Conclusion

Radiation‐induced brain injury remains a critical challenge for patients undergoing treatment for brain tumors, often resulting in cognitive decline and reduced quality of life. Autophagy, a central mechanism for maintaining cellular homeostasis, plays a dual role in this context. While autophagy can protect neurons by clearing damaged components and reducing inflammation, its excessive activation may worsen brain injury. Therapeutic strategies targeting autophagy regulators, such as mTOR inhibitors and AMPK activators, show potential in mitigating radiation‐induced damage. Furthermore, combination therapies that integrate autophagy modulation with antioxidants, anti‐inflammatory agents, or BBB stabilizers may enhance therapeutic efficacy. However, achieving an optimal balance between protective and detrimental autophagy remains a critical challenge. Precise optimization of dosage and timing is essential to avoid cytotoxicity associated with autophagy overactivation. The safety profiles of autophagy modulators, particularly in vulnerable populations such as pediatric patients, warrant rigorous preclinical and clinical evaluation. Additionally, the heterogeneity of radiation‐induced brain injury—shaped by factors such as radiation dose, fractionation schedules, and individual genetic susceptibility—calls for personalized approaches to autophagy‐based interventions. Continued research is needed to elucidate the role of autophagy across different phases of brain injury and its effects on various neural regions. Such insights are vital to ensure that future therapies are both safe and effective.

In summary, while significant progress has been made in understanding autophagy's role in radiation‐induced brain injury, more work is needed to fully harness its therapeutic potential and improve patient outcomes.

## Author Contributions

C.Z. devised the review. J.T., Y.M., and Y.W. reviewed the literature and wrote the manuscript drafts. Y.W., D.L., T.L., L.S., Y.M., and C.Z. carried out writing‐review and editing. Y.W. and C.Z. substantially contributed to the literature review and the writing of this manuscript. All authors have read and agreed to the published version of the manuscript.

## Conflicts of Interest

The authors declare no conflicts of interest.

## Data Availability

All the data supporting the findings of this study are included in this article.
